# Unexpected carboxyhemoglobin half-life during cardiopulmonary resuscitation: a case report

**DOI:** 10.1186/s12245-023-00492-2

**Published:** 2023-03-21

**Authors:** Nicolas Delvau, Andrea Penaloza, Véronique Franssen, Frédéric Thys, Pierre-Marie Roy, Philippe Hantson

**Affiliations:** 1 Departments of Emergency Care, Cliniques Universitaires Saint-Luc, Université Catholique de Louvain, 1200 Brussels, Belgium; 2 Emergency Department, GHDC: Grand Hopital de Charleroi, 6000 Charleroi, Belgium; 3 Emergency Department, CHU Angers: Centre Hospitalier Universitaire d’Angers, Angers Cedex 01, 49033 Angers, France; 4 Departments of Intensive Care, Cliniques Universitaires Saint-Luc, Université Catholique de Louvain, 1200 Brussels, Belgium

**Keywords:** Carbon monoxide poisoning, Cardiac arrest, Cardiopulmonary resuscitation, Carboxyhemoglobin half-life, Case report

## Abstract

**Background:**

Cardiac arrest (CA) following CO poisoning (CO-induced CA) exposes patients to an extremely high risk of mortality and remains challenging to treat effectively. Terminal carboxyhemoglobin elimination half-life (COHbt_1/2_) is critically affected by ventilation, oxygen therapy, and cardiac output, which are severely affected conditions in cases of CA.

**Case presentation:**

Asystole occurred in an 18-year-old woman after unintentional exposure to CO in her bathroom. Cardiopulmonary resuscitation (CPR) was started immediately, including mechanical ventilation with a fraction of inspired oxygen (FiO_2_) of 1.0 and external chest compressions with a LUCAS® device. CPR was stopped after 101 min, as it was unsuccessful. During this period, we calculated a COHbt_1/2_ of 40.3 min using a single compartmental model.

**Conclusions:**

This result suggests that prolongation of CPR time needed to back COHb at 10%, a level more compatible with successful return of spontaneous circulation (ROSC), could be compatible with a realistic CPR time. Calculating COHbt_1/2_ during CPR may help with decision-making regarding the optimal duration of resuscitation efforts and further with HBO_2_ or ECLS. Further evidence-based data are needed to confirm this result.

## Background

Carbon monoxide (CO) poisoning remains the main cause of unintentional, intentional, and fire-related poisoning deaths around the world [1–3]. The overall mortality rate is marginally affected by the level of medical care provided, with a fatality rate of approximately 3% [4]. Commonly identified factors that are strongly correlated to a higher mortality rate include concomitant cyanide intoxication, loss of consciousness (LOC), COHb levels > 25%, arterial pH, and the need for endotracheal intubation. Moreover, the mortality rate of CO-induced CA may be as high as 100% [5]. However, in certain cases, some patients with CO poisoning could benefit from more aggressive or prolonged treatment [6].

Current evidence shows that oxygen (O_2_) therapy reduces blood carbon monoxide elimination half-life (COHbt_1/2_), but its influence on neurological outcome is inconsistent [7–11]. Despite a lack of high-level evidence, hyperbaric oxygen (HBO_2_) therapy is recommended by many scientific societies for severe CO poisoning [12–15]. There are currently no specific recommendations that deal specifically with cardiac arrest (CA) following CO poisoning (CO-induced CA) [16–18]. A review conducted in 2001 concluded that there was an extremely poor survival prognosis for CO-induced CA, with or without HBO_2_ [5]. To our knowledge, there have been no more recent publications on this topic, and none of the studies mentioned COHbt_1/2_ during CPR. COHbt_1/2_ is affected by ventilation, oxygen therapy, and cardiac output, all of which are conditions severely impaired by CA [11, 19]. As such, we investigated COHbt_1/2_ during prolonged CPR in a case of CA following unintentional CO exposure.

## Case presentation

**Fig. 1:**
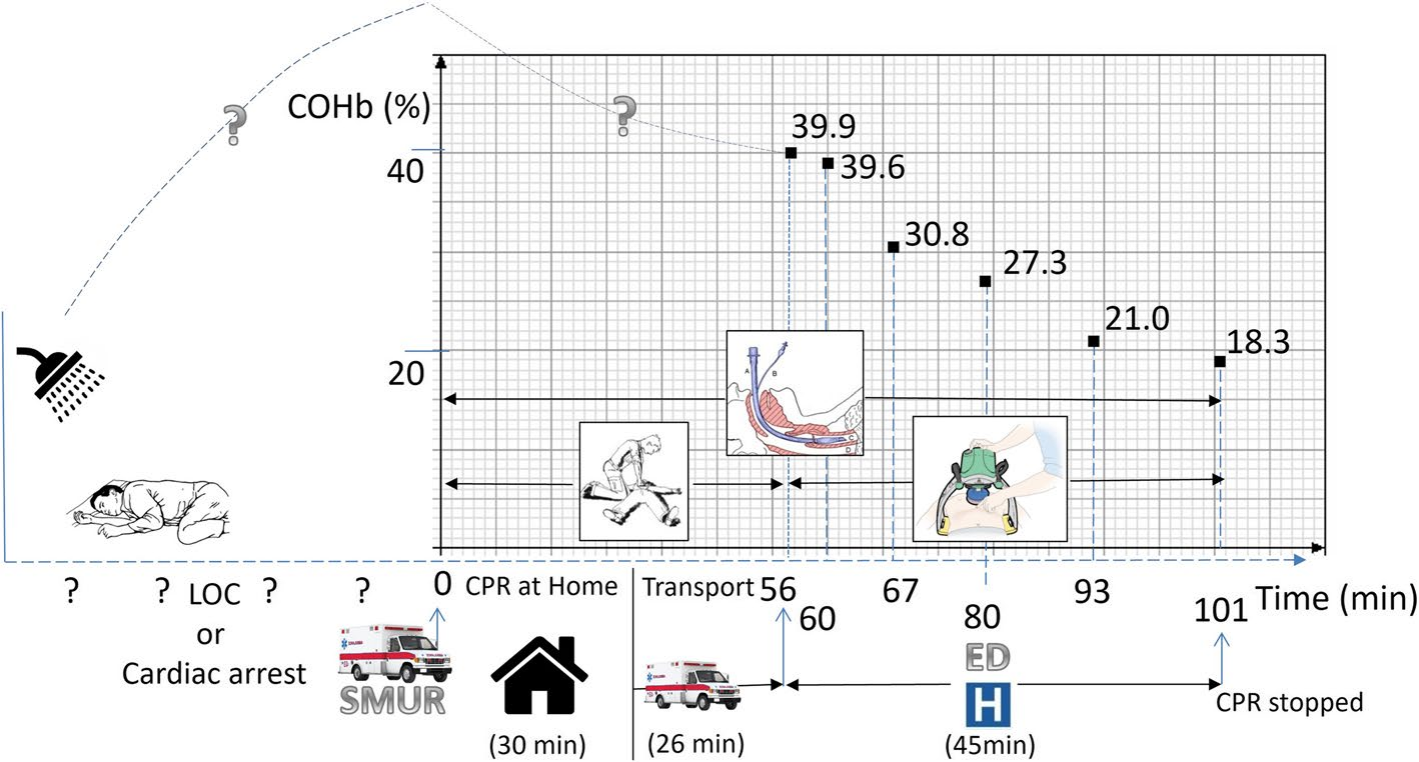
Timeline of our case report on cardiac arrest following CO poisoning and COHbt_1/2_. LOC, loss of consciousness; CPR, cardiopulmonary resuscitation; LUCAS® external chest compressions device for the last 45 min; SMUR, mobile emergency and resuscitation service

An 18-year-old woman with no previous medical history was unintentionally exposed to carbon monoxide (CO) from a water heater at home and was discovered unconscious on her bathroom floor. According to her family, she had gone into the bathroom 1 h earlier. The mobile emergency and resuscitation service (SMUR) found her pulseless in asystole and CO poisoning was confirmed via a bedside CO detector (alarm without a displayed value) (Fig. 1). Advanced cardiac life support (ACLS) was applied at home by the emergency physician according to European Resuscitation Council (ERC) guidelines [20]. Intubation was immediately performed on site and mechanical ventilation was initiated with a fraction of inspired oxygen (FiO_2_) of 1.0 for the entire duration of resuscitation (Fig. 1). Following evidence of unsuccessful CPR after 30 min with sustained asystole, the decision was made to transport the patient to a tertiary referral hospital while ongoing CPR was performed.

CPR was continued when the patient arrived at the referral hospital. During CPR, repeated arterial blood gas analysis was performed using a point-of-care ABL90Flex® blood gas analyzer (Radiometer™, Radiometer Medical Aps, Denmark). Our secondary focus for the six blood samples analyzed during CPR was arterial COHb. In total, the unsuccessful resuscitation efforts lasted for 101 min. The patient was ventilated with a FiO_2_ of 1.0 during this period and chest compressions were performed with an external LUCAS® thoracic compression device (Fig. 1) during the last 45 min of CPR. Assuming that the elimination of CO followed a single compartmental model, we calculated a COHbt_1/2_ of 40.3 min from the six data points [11].

## Discussion and conclusions

In this patient with CO-induced CA, we observed a COHbt_1/2_ of 40.3 min during CPR with external chest compressions and controlled mechanical ventilation with an FiO_2_ of 1.0. This was much lower than expected and closer to the figure reported with HBO_2_ therapy with spontaneous breathing [11, 21–24]. This finding suggests that, in cases of CO-induced CA, the duration of resuscitation may be extended up to the conventional recommendation of 30 min. This is with an empirical objective of reaching a COHb level lower than the 10% typically found in smokers and considered as a non or low COHb level toxicity [20, 25].

For patients with spontaneous breathing with normobaric oxygen therapy and a non-rebreathing mask, COHbt_1/2_ is 74 ± 25 min [26 to 148] [11]. For normobaric oxygen therapy, Levasseur et al. reported a COHBt_1/2_ of 92 ± 40 min in fire victims with controlled ventilation with a FiO_2_ of 1.0 [21]. Conversely, with HBO_2_ therapy, the COHbt_1/2_ is estimated to be around 23.3 min or even > 43 min [22, 26]. However, these values were assessed in the absence of severe cardiopulmonary dysfunction and may be underestimated in cases of CA [26]. It seems that no other case report regarding COHb_t1/2_ in a pure CO-poisoning and CA patient has been published. The reasons for this unexpectedly short CO half-life during CPR merit discussion. First, we can hypothesize that it is, at least partly, due to early intubation and mechanical ventilation with a FiO_2_ of 1.0 and sustained external chest compressions. Second, COHb_t1/2_ in clinical practice is typically quoted on normobaric oxygen therapy at 74 min ± 25 [26–148], which reflects a very wide range that includes our low value [11]. In fact, the elimination time of exposure to CO for a short duration may enable lower CO diffusion to extravascular compartments. For a short duration at high-level exposure, COHb_t1/2_ is shorter than a long duration at low-level exposure [27]. In addition, COHb_t1/2_ may be slightly shorter for females due to total hemoglobin and muscle myoglobin mass [28, 29]. Furthermore, despite the fact that we applied the ERC guidelines [20], arterial pH was less than 7.0 during CPR. With this acidity level, we suspect a lower affinity of Hb for CO, even if we did not confirm this hypothesis in a recent meta-analysis [30].

High COHb levels are a marker of exposure but unrelated to clinical presentation or outcome [14]. Even in the absence of strong evidence, we suggest that COHb levels should be monitored regularly during CPR with the objective of reaching COHb levels lower than 10% before deciding to stop CPR. Indeed, COHb levels lower than 10% are frequently observed in smokers and are considered to be non-toxic [25]. This target could be reached within a reasonable period compatible with ROSC [31]. However, CO binds to cardiomyoglobin and to cytochrome-c oxidase, which interferes with cellular respiration [32, 33], but is not available in clinical practice to estimate the CO body burden. Thus, the CO burden impact could be prolonged beyond %COHb back to 10% [27, 34].

Unfortunately, the patient did not survive. We stopped CPR without reaching the COHb level at 10% because the “no flow” before CPR was considered too long to hope for a CPR success. This adverse outcome is not surprising [5]. The current survival rate of all-cause out-of-hospital CA is less than 10% [35]. In cases of CO-induced CA, brain injury is worsened by the absence of O_2_ delivery and by the other biochemical mechanisms of CO toxicity [36]. After O_2_ supply therapy, mitochondria respiration inhibited by CO is restored more slowly compared to mitochondria simply deprived of oxygen [5]. These mechanisms lead to consider HBO_2_ therapy for CO-induced CA. Hampson et al. reported that 100% of the directors of HBO_2_ therapy medical centers would recommend HBO_2_ therapy for extremely unstable CO poisoning, predicting a 74% chance of survival after HBO_2_, and a 28% chance of complete recovery without neurologic sequelae [5]. However, in Hampson’s case series, the mortality rate among 18 patients with CO-related CA treated by HBO_2_ was 100% [5]. Indeed, patients who developed CO-induced CA rarely survived, even if a return of spontaneous circulation (ROSC) was achieved [37]. No study considers the COHb% threshold in a protocol to assess the chances of survival. Ideally, HBO_2_ therapy should be initiated as soon as possible in the first 6 h. Six hours corresponds to the “golden hour” that could decrease the incidence of delayed neurological sequelae from CO poisoning [38]. Unstable patients requiring multiple vasoactive drugs are not easily transferred safely to an HBO_2_ chamber. Despite this, some authors suggest speeding up the transfer to HBO_2_ chambers due to the fear of impending cardiac arrest [39]. It should be noted that patients who require chest compressions, intubation, or positive pressure ventilation during their resuscitation are at an increased risk of an HBO_2_-induced tension pneumothorax [39]. On the other hand, extra-corporeal life support (ECLS) has been applied with success in some selected cases as during cardiovascular failure but not after cardiac arrest [40–42]. ECLS was not pursued because in our Center ECLS protocol is only considered in cardiac arrest with a certainty of less than 10 min “no flow”. It appears that ECLS and HBO_2_ may have complementary effects: ECLS improves oxygenation to target organs independently from heart and lung failure, while HBO_2_ is more effective in ensuring high O_2_ dissolution in brain and heart cells [43]. The 2015 guidelines of the European Resuscitation Council concluded that there is an unproven benefit of transporting patients with ROSC to an HBO_2_ therapy facility and that such a decision must be considered on a case-by-case basis [37]. Transfer to a tertiary referral hospital could be partly motivated by the possible consideration of organ donation [44, 45].

The vast majority (88%) of patients who achieved sustained ROSC after CA from any cause did so within 30 min of CPR, and CPR longer than 20 min after asystole is generally accepted as futile [46]. However, in specific cases of presumably reversible causes (airway obstruction, hypothermia, poisoning, etc.), the optimal CPR duration remains a case-by-case bedside decision that may, for CO-induced CA, take into account the rate of CO elimination [46, 47]. Interestingly, in Hampson’s fatal CO poisoning retrospective review, the duration of CPR ranged from 19 to 45 min [5]. The lack of information about the duration of the “no flow” period could also affect the physician’s attitude to the prolongation of CPR. The difference between patients with signs of life before they go into cardiac arrest and those already found with no pulse is an important factor to consider in assessing hypoxic brain injury. Indeed, we only found two clinical descriptions of patient survival with good neurological recovery for patients that underwent mechanical ventilation and hypothermic therapy, without HBO_2_ therapy. Both patients presented with CA in the emergency department (ED). ROSC was achieved after a few minutes without a “no flow” period [48–50]. The determination of the “no flow” period appears to be particularly difficult in patients with CO poisoning as a loss of consciousness may precede CA and may be erroneously considered an absence of cerebral blood flow. In our opinion, CPR should be initiated in any case of CO-induced CA and extended when circulatory collapse or CA is witnessed by responders. CPR could be prolonged until a COHb rate < 10% is reached, a level expected to allow ROSC occurrence, especially if the CO body burden is suspected to be low in case of short CO exposure time.

In our case report of CO-induced CA with prolonged CPR including controlled mechanical ventilation at a FiO_2_ of 1.0, we observed a COHbt_1/2_ much lower than expected. This result suggests that prolonged CPR may lead to a COHb level which could be compatible with successful ROSC. Calculating COHbt_1/2_ during CPR may help in decision-making regarding the optimal duration of resuscitation efforts and further therapy with HBO_2_ or ECLS. Further evidence-based data are needed to confirm this result.

## Data Availability

Not applicable (see Fig. 1).

## Abbreviations

ACLS: Advanced cardiac life support
CA: Cardiac arrest
CO-induced CA: Cardiac arrest following CO poisoning
CO: Carbon monoxide
CPR: Cardiopulmonary resuscitation
ECLS: Extra-corporeal life support
ED: Emergency department
ERC: European Resuscitation Council
FiO_2_: Fraction of inspired oxygen
HBO_2_: Hyperbaric oxygen
COHb: Carboxyhemoglobin
COHbt_1/2_: Carbon monoxide elimination half-life
LOC: Loss of consciousness
O_2_: Oxygen
ROSC: Return of spontaneous circulation
SMUR: Mobile emergency and resuscitation service
